# Robotic surgery versus laparoscopic surgery for rectal cancer: a comparative study on surgical safety and functional outcomes

**DOI:** 10.1111/ans.19302

**Published:** 2024-11-11

**Authors:** Li TengTeng, Fu HaiXiao, Fu Wei, Zhang Xuan

**Affiliations:** ^1^ Department of General Surgery The Affiliated Hospital of Xuzhou Medical University Xuzhou Jiangsu Province China

**Keywords:** comparative study, functional outcomes, laparoscopic surgery, rectal cancer, robotic surgery, surgical safety

## Abstract

**Backgrounds:**

This study aims to evaluate the clinical efficacy and functional outcomes of DA Vinci (Xi)‐assisted surgery compared to conventional laparoscopic surgery for middle and low rectal cancer, focusing on oncologic cure and functional preservation.

**Methods:**

Between December 2020 and June 2021, 102 patients with middle and low rectal cancer (tumour lower margin ≤10 cm) were enrolled at the affiliated Hospital of Xuzhou Medical University. Participants were divided into two groups: robot‐assisted (*n* = 51) and laparoscopy‐assisted (*n* = 51). Each group underwent a radical resection using their assigned method. Clinical and functional outcomes were analysed post‐surgery.

**Results:**

Preoperative data did not differ significantly between groups (*P* > 0.05). All surgeries were successfully completed without conversion to open surgery. The robotic group experienced significantly less intraoperative blood loss (55.2 ± 29.8 mL vs. 109.5 ± 58.5 mL) and faster recovery in gastrointestinal function (35.1 ± 9.4 h vs. 40.7 ± 1.9 h), diet recovery (2.1 ± 0.8 days vs. 2.9 ± 0.4 days), and catheter removal (2.9 ± 2.7 days vs. 5.3 ± 2.1 days). The robotic group also dissected more lymph nodes (23 ± 6 vs. 15 ± 4). However, they had longer operative times (239.8 ± 29.6 min vs. 141.1 ± 18.5 min) and higher hospital costs. Satisfaction levels regarding defecation, voiding, and sexual functions were notably higher in the robotic group.

**Conclusion:**

No significant differences in surgical safety or immediate postoperative outcomes were observed between robotic and laparoscopic approaches. However, robotic surgery demonstrated superior lymph node dissection, anal function preservation, and gastrointestinal recovery, enhancing overall functional outcomes.

## Graphical abstract

In this study, surgery was performed using this procedure‐based and close follow‐up showed that robotic surgery showed corresponding advantages over conventional laparoscopic resection in terms of protection of the pelvic autonomic nerves and achieved better late results.

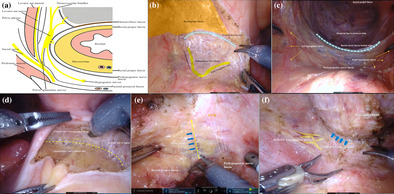

## Introduction

In rectal cancer surgery, oncological cure and function preservation are the primary objectives. There has been a notable improvement in local recurrence rates and tumour‐specific survival after total mesorectal excision (TME).^1^ Even experienced colorectal surgeons worry about sexual and urinary function outcomes.^2^ In rectal cancer patients post‐TME, urinary dysfunction occurs in 0–27% and sexual dysfunction in 11–55% of cases.^3^ Preoperative radiotherapy and adjuvant chemotherapy may negatively affect postoperative function, but intraoperative nerve injury is the primary cause of sexual and urological dysfunction.^4^ Preserving sexual and urinary function in young patients requires satisfactory visualization and appropriate traction and countertraction.^5^

Surgical robots offer a stable three‐dimensional view, tremor filtering, and ergonomic instruments, enabling surgeons to achieve fine dissection and observe the surgical site at higher magnification and from multiple perspectives.^6^, ^7^ Robotic systems are extensively used in pelvic surgery, and robotic total mesorectal excision (TME) for rectal cancer has shown equivalent short‐term oncological outcomes to traditional open and laparoscopic approaches.^8^ It is controversial whether minimally invasive techniques, such as laparoscopy, are superior to open total mesorectal excision (TME), when it comes to urological and sexual dysfunction.^9^ Our findings indicate that three‐dimensional magnification and a flexible robotic arm can help preserve pelvic autonomic nerves, which improves sexual and urinary function. Successful pelvic autonomic nerve preservation (PANP) depends on understanding pelvic nerve anatomy, lymphatic metastasis patterns, and careful attention to anatomical layers during surgery.

## Materials and methods

### Inclusion criteria of cases

(i) age >18 years; (ii) colorectal adenocarcinoma confirmed by colonoscopy biopsy; (iii) tumour distance from anal margin <10 cm; (iv) no distant metastasis by ultrasound and CT; (v) pelvic MRI or transrectal ultrasound showing CT_1–3_ N_0–1_ or undergoing neoadjuvant radiotherapy ypT_1–3_; (vi) no history of other malignancies; (vii) suitable for laparoscopic and robotic surgery; (viii) patient informed consent.

### Exclusion criteria

Included cT_1_N_0_ suitable for local resection, emergency operations for acute intestinal issues (obstruction, bleeding, perforation), multiple primary colorectal malignancies, familial adenomatous polyposis, Lynch syndrome, inflammatory bowel disease, simultaneous colectomy, ASA classification >Class III, pregnancy, and lactation. The study conditions and purpose were not communicated to patients or their families.

### Subjects and groups

Between December 2020 and June 2021, 102 patients with middle and low rectal cancer at the Affiliated Hospital of Xuzhou Medical University underwent radical resection, with 51 assigned to robot‐assisted and 51 to laparoscopy‐assisted groups, all with informed consent and ethical approval (Ethics Review Number XYFY 2020‐JS003‐03).

### Surgery methods

The robotic surgery group adhered to the ‘Chinese Expert Consensus on Robotic Surgery for Colorectal Cancer (2020)’ for surgical posture and trocar positioning, and followed the 2018 guidelines for laparoscopic radical resection of rectal cancer.^10^, ^11^

**Fig. 1:**
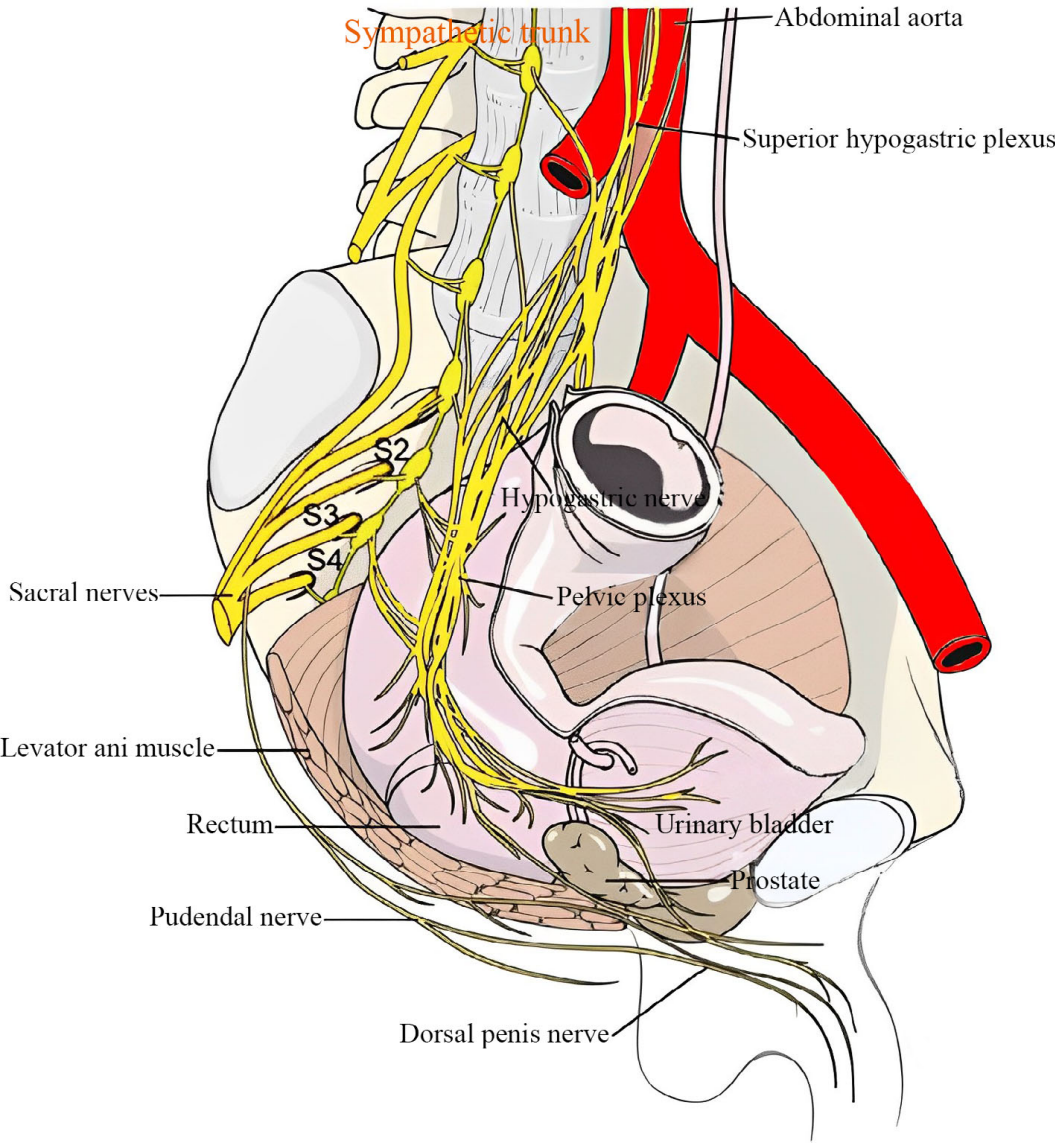
The close relationship between sexual and urinary function and the pelvic autonomic plexus and neurovascular bundle in robotic‐assisted radical rectal cancer surgery. As illustrated in Fig. 2, a stereotyped, flow‐based surgical technique was used to preserve the rectal tract fascia.

**Fig. 2:**
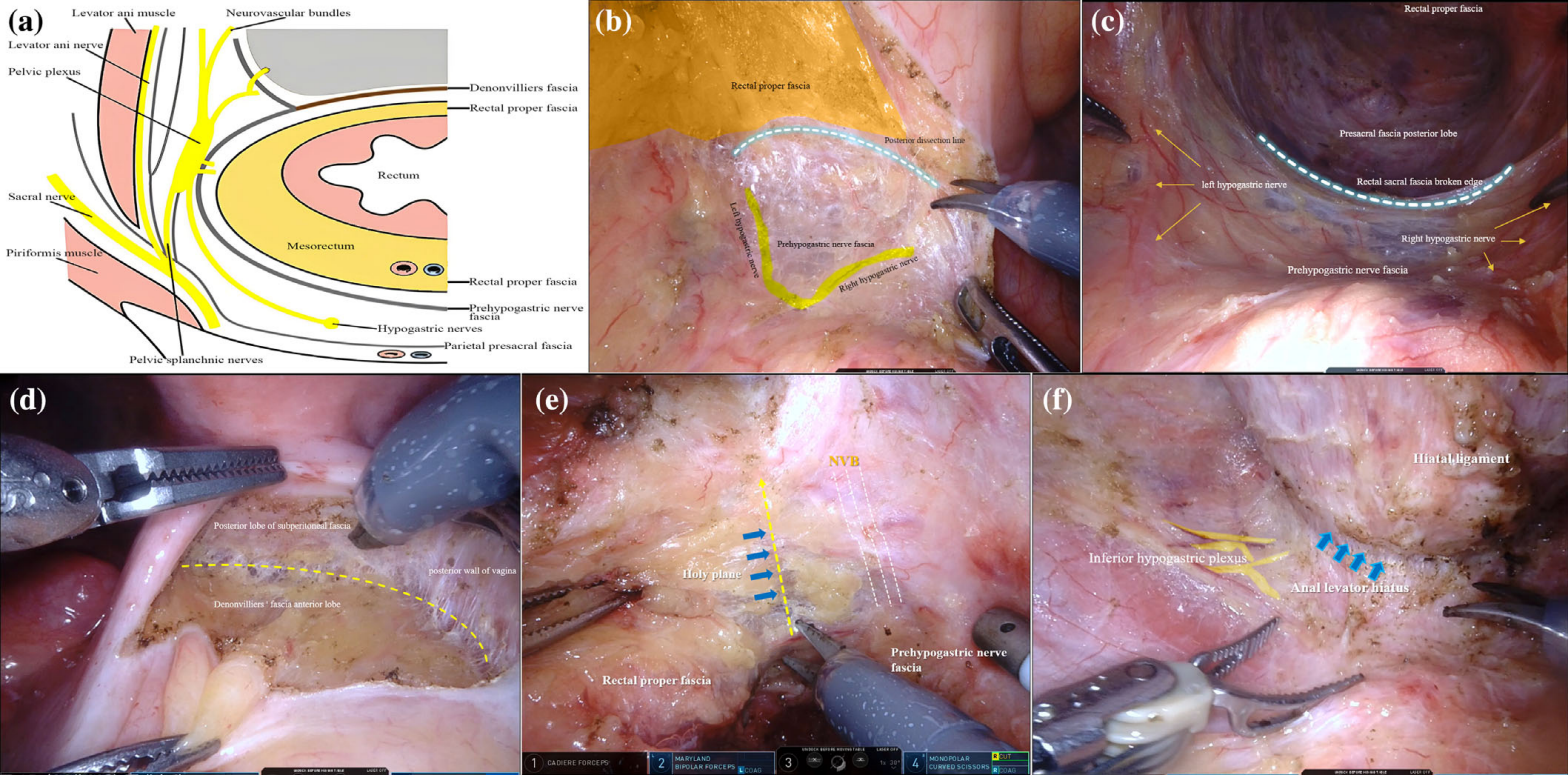
(a) Circumferential structures of the rectum. (b) First step: posterior pelvic dissection. (c) The involves a deep posterior pelvic dissection. (d) The third step involves the dissection of the anterior pelvic region. (e) Fourth step: posterolateral and anterolateral pelvic dissection. (f) Last deeper pelvic dissection toward the pelvic floor in the fifth step.

Figure 1 illustrates the close relationship between sexual and urinary function and the pelvic autonomic plexus and neurovascular bundle in robotic‐assisted rectal cancer surgery, while Fig. 2 presents a flow‐based surgical technique designed to preserve these neural structures and maintain rectal fascia integrity, addressing the risks of nerve injury to various pelvic nerves during total mesorectal excision (TME).

### Crucial measures for safeguarding nerve function

(i) First step: posterior pelvic dissection (Fig. 2b). The superior hypogastric plexus is prone to injury during high ligation at the root of the inferior mesenteric artery (IMA). (ii) During deep posterior pelvic dissection, the hypogastric nerves are vulnerable to injury. However, following the correct fascial planes, such as the prehypogastric nerve fascia, significantly reduces this risk (Fig. 2c). (iii) The third step involves dissecting the anterior pelvic region, separating the peritoneal reflection to reveal the seminal vesicle in men and the vaginal wall in women. Dissection should proceed below the Denonvilliers fascia and laterally along the seminal vesicles for optimal nerve preservation (Fig. 2d). (iv) Fourth step: posterolateral and anterolateral pelvic dissection (Fig. 2e). (v) The fifth step involves deeper pelvic dissection towards the pelvic floor (Fig. 2f).

## Collection of data and observation indicators

### General data

The study variables include age, sex, BMI, abdominal surgery history, ASA grade, ECOG score, tumour‐to‐anal margin distance, pathological diagnosis, cTNM stage, preoperative tumour markers (CEA, AFP, CA19‐9), and neoadjuvant chemotherapy.

### Preoperative indicators

This study evaluated operation method, preventive stoma use, duration, intraoperative blood loss, transfusion need, conversion to laparotomy, time to first bowel movement and feeding, catheter removal timing, length of hospital stay, pain severity, postoperative complications (Clavien‐Dindo classification), hospitalization costs, and sphincter preservation rate.

### Postoperative pathological parameters

This study assessed tumour length, resected specimen length, margin status, TME integrity, tumour differentiation, lymph node count, circumferential margin, TNM staging (eighth edition).

### Functional indicators

Defecation function was assessed using the Wexner score and LARS score, while voiding function was evaluated with the IPSS, including nocturnal urination; male sexual function was measured by the modified IIEF‐15, and female sexual function was assessed with the FSFI‐19.

### Follow‐up methods

Patients were followed every 3 months until December 2022 through outpatient visits or telephone, with assessments including blood tests, tumour markers, imaging, colonoscopy for recurrence and metastasis detection, and evaluation of autonomic and pelvic nerve function using urination, defecation, and sexual function scales.

### Statistical analyses

Statistical analysis was conducted using SPSS 24.0 software. Normally distributed data were expressed as x¯ ± s and compared using independent samples *t*‐test. Non‐normally distributed data were expressed as M (range) and compared using the Mann–Whitney *U*‐test. Categorical data were expressed as percentages and compared using χ^2^ test or Fisher's exact test. Ordinal data were compared using the Mann–Whitney *U*‐test.

## Results

### Patient characteristics

Table 1 shows no significant differences between the two groups in age, gender, BMI, surgical history, ASA grade, ECOG score, tumour location, pathological diagnosis, cTNM stage, preoperative tumour marker levels, and neoadjuvant chemotherapy proportion.

**Table 1:** Comparison of the general data between the two groups (mean ± SD)

Patient characteristics	R‐TME (*n* = 51)	L‐TME (*n* = 51)		*P*‐value
Gender, no. (%)				0.685
Male	30 (58.8)	32 (62.7)		
Female	21 (41.2)	19 (37.3)		
Age	53 ± 6	54 ± 4		0.751
BMI (kg/m^2^)	25 ± 3	25 ± 3		0.777
Prior abdominal surgery (*n* (%))	5 (9.8%)	4 (7.8%)		1.000
ASA score, no. (%)				0.953
I	24 (47.1%)	23 (45.1%)		
II	19 (37.2%)	21 (41.2%)		
III	8 (15.7%)	7 (13.7%)		
ECOG				0.725
0 point	22 (43.1%)	20 (39.3%)		
1 point	25 (49.1%)	27 (52.9%)		
2 points	4 (7.8%)	4 (7.8%)		
Distance from the anal verge (cm)	6.0 ± 2.4	5.8 ± 2.6		0.553
Histological type of carcinoma (*n* (%))				0.767
Adenocarcinoma	45 (88.2%)	44 (86.3%)		
Mucinous adenocarcinoma	6 (11.8%)	7 (13.7%)		
cTNM stage (*n* (%))				0.661
I	4 (7.8%)	6 (11.8%)		
II	30 (58.8%)	29 (56.9%)		
III	17 (33.4%)	16 (31.3%)		
Preoperative CEA (ng/mL, *M* (range))	4.1 (0.9 ~ 149.0)	4.9 (0.8 ~ 151.7)		0.436
PreoperativeCA19‐9 (U/mL, *M* (range))	8.2 (0.6 ~ 56.9)	9.2 (0.6 ~ 65.4)		0.947
Neoadjuvant chemotherapy, no. (%)	5 (9.8%)	6 (11.8%)		0.750

### Comparison of preoperative indexes between the two groups

The robot and laparoscopy groups showed significant differences in intraoperative blood loss, operation duration, first bowel movement, oral intake time, catheter removal time, postoperative pain, and treatment costs (*P* < 0.05). However, no significant differences were found in prophylactic colostomy, conversion to laparotomy, intraoperative blood transfusion, time to ambulation, and postoperative complications (*P* > 0.05). Anastomotic leakage was treated with irrigation, drainage, and anti‐infective therapy. Intestinal adhesion was managed with decompression (Table 2).

**Table 2:** Surgical data

	R‐TME (*n* = 51)	L‐TME (*n* = 51)		*P‐*values
Type of operation, no. (%)				0.681
Low anterior resection	40 (78.4%)	37 (72.5%)		
Mile's	7 (13.7%)	11 (21.6%)		
Lateral lymph node dissection	4 (7.8%)	3 (5.9%)		
Bacon	4 (7.8%)	3 (5.9%)		
Protective ileostomy, no. (%)	8 (15.7%)	9 (17.6%)		0.791
Operative time (min)	239.8 ± 29.6	141.1 ± 18.5		0.000
Estimated blood loss (mL)	55.2 ± 29.8	109.5 ± 58.5		0.000
Blood transfusions (n (%))	0 (0.0)	0 (0.0)		
Conversion to open surgery	0 (0.0)	0 (0.0)		
Days to first flatus emission after surgery (h)	35.1 ± 9.4	40.7 ± 1.9		0.003
First flow time (d)	2.1 ± 0.8	2.9 ± 0.4		0.000
Time of catheter removal (d)	2.9 ± 2.7	5.3 ± 2.1		0.000
Time to get out of bed	1.41 ± 0.3	1.54 ± 0.1		0.257
Complications, no. (%)	6 (11.8%)	7 (13.7%)		0.767
Anastomotic leakage	2	2		
Anastomotic bleeding	0	0		
Presacral infection	1	1		
Wound infection	0	0		
Pulmonary infection	2	2		
Complications of stoma	0	0		
Intestinal adhesion, ileus	1	1		
Small intestinal leakage	0	0		
Urinary retention	0	1		
Clavien–Dindo grade, no. (%)				0.767
I	3	4		
II	3	3		
III	0	0		
Postoperative pain grading, no. (%)				0.004
Level 1	13 (25.4%)	6 (11.8%)		
Level 2	15 (29.4%)	7 (13.7%)		
Level 3	17 (33.4%)	26 (50.9%)		
Level 4	6 (11.8%)	12 (23.6%)		
Postoperative hospital stay (d)	8.2 ± 2.1	8.3 ± 2.7		0.832
Hospitalization expenses (10 000 yuan)	7.1 ± 4.5	6.7 ± 1.6		0.001
Mortality within 30 days of operation, no. (%)	0 (0.0)	0 (0.0)		–
Sphincter preservation rate, no. (%)	86.2%	78.4%		0.299

### Pathological outcomes

A significant difference was found in the number of lymph node dissections between the robot and laparoscopy groups (*P* < 0.05). No significant differences were observed in tumour length, resection length, residual tumour tissue, margin distances, TME integrity, circumferential margin, postoperative pathological stage, and differentiation degree (Table 3).

**Table 3:** Pathological data

Pathology outcomes	R‐TME (*n* = 51)	L‐TME (*n* = 51)		*P‐*values
Tumour size (cm)	3.8 ± 1.1	3.7 ± 1.0		0.088
Specimen excision length (cm)	18.7 ± 3.2	18.3 ± 2.8		0.201
Distance of upper incisal margin of tumour (cm)	11.8 ± 3.6	10.2 ± 2.7		0.104
Distal resection margin (cm)	2.7 ± 0.8	2.5 ± 0.6		0.271
Residual cancer tissue of margin, no. (%)	0 (0.0)	0 (0.0)		–
TME grading, no. (%)				0.713
Complete	48 (94.1%)	46 (90.2%)		
Near complete	3 (5.9%)	5 (9.8%)		
Incomplete	0 (0.0)	0 (0.0)		–
Tumour grade of differentiation				0.675
Well‐differentiated	4 (7.8%)	6 (11.8%)		
Moderately differentiated	41 (80.4%)	39 (76.4%)		
Poorly differentiated	6 (11.8%)	6 (11.8%)		
No. harvested lymph nodes	23 ± 6	15 ± 4		0.000
Positive CRM, no. (%)	0	1 (1.9%)		1.000
Mesenteric destruction, no. (%)	0 (0.0)	1 (1.9%)		1.000
pTNM stage, no. (%)				0.798
I	6 (11.8%)	5 (9.8%)		
II	17 (33.4%)	20 (39.3%)		
III	28 (54.8%)	26 (50.9%)		
Adjuvant chemo, no. (%)	35 (68.6%)	36 (70.6%)		0.830

### Comparison of follow‐up between the two groups

Both groups had complete follow‐up with no significant differences in postoperative colostomy, local recurrence, distant metastasis, and death (*P* > 0.05), but robotic TME demonstrated faster recovery in erectile and urinary function, with significant differences in Wexner, LARS, IPSS, nocturnal urination, FIFS, and IIEF scores at 3, 6, 9, and 12 months; erectile function recovered in 6 months with robotic TME versus 12 months with laparoscopic TME (Table 4).

**Table 4:** Comparison of postoperative follow‐up

	R‐TME (*n* = 51)	L‐TME (*n* = 51)		*P‐*values
Stoma reversals, no. (%)	8	8		0.790
Local Recurrence, no. (%)	0	0		‐
Distant metastases, no. (%)	0	0		‐
Wexner				
Before surgery	0	0		‐
3 months after surgery	2.08 ± 0.29	3.66 ± 0.62		0.000
6 months after surgery	2.42 ± 0.32	3.62 ± 0.28		0.000
9 months after surgery	1.89 ± 1.10	2.33 ± 1.01		0.040
12 months after surgery	1.34 ± 0.11	1.99 ± 1.33		0.001
LARS				
Before surgery	12.25 ± 1.08	12.60 ± 1.11		0.111
3 months after surgery	21.15 ± 2.04	24.18 ± 6.04		0.002
6 months after surgery	20.92 ± 2.85	22.90 ± 5.77		0.030
9 months after surgery	18.94 ± 3.62	21.24 ± 6.09		0.023
12 months after surgery	18.11 ± 3.54	20.85 ± 6.19		0.007
IPSS				
Before surgery	4.43 ± 0.33	4.56 ± 0.36		0.078
3 months after surgery	6.13 ± 0.45	7.77 ± 1.57		0.001
6 months after surgery	5.53 ± 0.57	7.02 ± 2.10		0.000
9 months after surgery	5.33 ± 0.39	6.72 ± 1.82		0.000
12 months after surgery	4.03 ± 0.26	6.43 ± 1.78		0.000
Nocturia				
Before surgery	0.49 ± 0.09	0.46 ± 0.07		0.147
3 months after surgery	1.83 ± 0.13	2.41 ± 0.08		0.000
6 months after surgery	1.56 ± 0.15	2.59 ± 0.18		0.000
9 months after surgery	1.06 ± 0.21	2.37 ± 0.86		0.000
12 months after surgery	1.08 ± 0.28	2.27 ± 0.23		0.000
FIFS				
Before surgery	24.07 ± 2.75	23.11 ± 2.77		0.120
3 months after surgery	4.37 ± 0.33	4.30 ± 0.21		0.206
6 months after surgery	15.27 ± 1.18	12.41 ± 1.14		0.000
9 months after surgery	20.43 ± 2.46	15.99 ± 2.08		0.000
12 months after surgery	22.63 ± 2.03	21.00 ± 2.73		0.001
IIEF				
Before surgery	65.84 ± 1.79	66.31 ± 1.73		0.180
3 months after surgery	5.75 ± 0.49	5.50 ± 0.89		0.073
6 months after surgery	37.78 ± 1.04	33.95 ± 9.47		0.005
9 months after surgery	46.77 ± 3.78	38.62 ± 7.66		0.000
12 months after surgery	38.57 ± 6.13	27.62 ± 8.20		0.000

## Discussion

Surgical intervention for rectal cancer aims to achieve oncologic cure while preserving function. Since the advent of total mesorectal excision (TME), local recurrence rates have significantly improved. Laparoscopic total mesorectal excision (TME) is a proven safe and effective treatment for rectal cancer.^12^, ^13^ Despite advancements in laparoscopic surgery, the complex anatomy and narrow pelvic cavity of the rectum pose significant technical challenges, leading to the increased use of DA Vinci robotic surgery, which enhances vision and precise manipulation, making it ideal for rectal cancer treatment.^14^ This study used a randomized controlled design to compare the safety and short‐term efficacy of robot‐assisted versus laparoscopic‐assisted radical resection for middle and low rectal cancer. Reported complication rates are 15.0% to 19.1% for robotic surgery and 18.4% to 26.2% for laparoscopic surgery.^8^, ^15^ This study found that postoperative complications occurred in 11.8% of patients undergoing robotic surgery, compared to 17.6% in the laparoscopic surgery group, consistent with Jayne *et al*.'s findings.^16^

The study found that intraoperative bleeding was significantly less in the R‐TME group compared to the L‐TME group, consistent with previous research.^17^ The robotic surgery system's enhanced visual field improves the identification of mesangial vessels and anatomical planes, reducing the risk of accidental blood vessel damage and bleeding. Its rotatable wrist instruments and stable manipulator ensure precise haemostasis in the narrow pelvic cavity, minimizing accidental injury to blood vessels.^18^ The robot surgery group showed faster exsufflation, quicker return to a fluid diet, and lower postoperative pain scores, consistent with multiple studies.^19^, ^20^ This outcome may be due to less irritation of non‐targeted intestines and faster recovery of gastrointestinal function after robotic colorectal surgery. The postoperative pathological examination showed a significant difference in the number of lymph nodes dissected between the robotic and laparoscopic groups. This can be attributed to the advanced visual assistance and operational platform provided by the robotic surgical system.^21^ Less intraoperative blood loss improves anatomical visibility, enabling precise dissection for lymph node removal and protection of nearby vessels and nerves. Furthermore, the incorporation of indocyanine green fluorescence imaging technology aids in identifying the lymphatic drainage areas of tumours, particularly when suspicious lymph nodes lie outside the traditional surgical field, thereby simplifying the lymphadenectomy process.^18^, ^19^ It is important to emphasize that a comprehensive and high‐quality lymphadenectomy during surgery serves as a crucial indicator for assessing the completeness of tumour resection and the long‐term oncological outcomes.

Studies pertaining to the radical resection of tumours have demonstrated that the utilization of robotic surgery can enhance the quality of total mesorectal excision (TME),20 and has some advantages in reducing the positive rate of CRM,^21^ but it still needs to be further verified.^22^, ^23^ The R‐TME exhibits similarities to L‐TME with regards to the distal resection margin's positive rate, local recurrence rate, and long‐term survival rate. Particularly, robotic techniques improve specimen quality and lower circumferential resection margin positivity in male patients with mid‐low rectal cancers, subsequently reducing local recurrences.^24^, ^25^ However, laparoscopic TME shows similar pathological, long‐term oncological, and functional outcomes when compared with robotic TME in female patients with rectal cancer. Presumably, these contrasting results are related to the pelvic anatomy differences between males (narrow pelvis, bulky mesorectum) and females (wider pelvis).^26^, ^27^ Robotic and laparoscopic surgeries showed no significant differences in specimen resection length, tumour lower cutting edge distance, surgical margin positivity rate, or postoperative hospital stay, with both R‐TME and L‐TME demonstrating equivalent radical effects; however, the robotic approach preserved the mesorectum in all cases compared to one instance of destruction in the laparoscopic group, attributed to the high‐definition three‐dimensional imaging of the robotic system.

The concept of tumour functional surgery has shifted rectal cancer treatment to not only focus on long‐term survival but also on enhancing post‐operative quality of life, particularly in terms of defecation, urination, and sexual function.^28^ Preserving pelvic autonomic nerves (PANP) improves genitourinary function post‐surgery but demands careful identification and protection of these nerves through precise anatomical techniques.^29^ Performing a total mesorectal excision (TME) with pelvic autonomic nerve plexus (PANP) preservation requires careful identification and protection of the pelvic nerves, with attention to areas prone to nerve damage. A survey of 74 patients who underwent robotic‐assisted radical resection for rectal cancer showed that satisfaction levels declined within 1‐month post‐surgery but returned to baseline after 1 year. Robotic surgery's enhanced visualization helps avoid nerve damage. A meta‐analysis of four studies involving 152 robotic and 161 laparoscopic rectal cancer patients found that robotic surgery significantly improved postoperative IIEF and IPSS scores. This study demonstrates that robotic surgery for radical tumour resection is safe and effective, significantly outperforming laparoscopic surgery in functional protection scores (Wexner, LARS, IPSS, IIEF, FIFS, and nocturia) and facilitating earlier recovery of defecation, urination, and sexual functions, with better preservation of sexual function noted in female patients.

This study acknowledges limitations such as a small sample size that may introduce biases, emphasizing the need for larger, multi‐centre trials with extended follow‐up to validate the DA Vinci robot's benefits; while initial operation times for robotic surgery are longer, they improve with proficiency and collaboration, though hospitalization costs are significantly higher due to equipment expenses; ultimately, robot‐assisted radical resection for middle and low rectal cancer proves as effective as laparoscopic surgery in the short term, with reduced intraoperative bleeding, faster gastrointestinal recovery, more lymph node dissection, better pelvic nerve protection, and no increase in surgical complications, while maintaining safety and enhancing the restoration of urinary, bowel, and sexual functions.

## Conflicts of interest

None declared.

## Funding information

This work was partially supported by the Science and Technology Development Fund Project of Affiliated Hospital of Xuzhou Medical University (XYFM2020042).

## Author contributions

**Li TengTeng:** Data curation; methodology; writing – original draft; writing – review and editing. **Fu HaiXiao:** Formal analysis; software; validation; writing – original draft. **Fu Wei:** Funding acquisition; project administration. **Zhang Xuan:** Conceptualization; supervision; writing – original draft; writing – review and editing.
